# The Upregulation of Two *CYP6* Genes Is Associated with the Response to Broflanilide in *Bactrocera dorsalis* (Hendel, 1912) (Diptera: Tephritidae)

**DOI:** 10.3390/insects17090950

**Published:** 2026-09-11

**Authors:** Bo Liu, Si-Yi Chen, Zhi-Hong Li, Shao-Kun Guo

**Affiliations:** 1 State Key Laboratory of Agricultural and Forestry Biosecurity, MARA Key Lab of Surveillance and Management for Plant Quarantine Pests, College of Plant Protection, China Agricultural University, Beijing 100193, China; 2018319010108@cau.edu.cn (B.L.); 2022319010111@cau.edu.cn (S.-Y.C.); lizh@cau.edu.cn (Z.-H.L.); 2 Sanya Institute, China Agricultural University, Sanya 572025, China

**Keywords:** *Bactrocera dorsalis*, broflanilide, cytochrome P450 monooxygenase, RNA interference, transcriptomics, insecticide susceptibility

## Abstract

*Bactrocera dorsalis* (Hendel) is a globally significant quarantine pest, and broflanilide represents a new insecticide with potential for its management. However, the molecular responses of *B. dorsalis* to broflanilide, particularly the involvement of cytochrome P450 genes, remain poorly understood. To address this gap, we compared the midgut and fat body transcriptomes of adults that survived 24 h of exposure to the median lethal concentration (LC_50_) of broflanilide with those of corresponding controls. Candidate P450 genes were further examined by quantitative real-time PCR (qRT-PCR), RNA interference (RNAi), and molecular docking. RNA-seq identified strong upregulation of *CYP6G8* (log_2_FC = 11.84 and 6.32) and *CYP6GX5* (log_2_FC = 6.48 and 7.83) in the broflanilide-exposed surviving group. qRT-PCR showed that *CYP6G8* was significantly upregulated in both tissues, whereas *CYP6GX5* was significantly upregulated in the fat body but showed a nonsignificant upward trend in the midgut. Silencing *CYP6G8* increased mortality from 43.21% to 64.14%, and *CYP6GX5* silencing increased mortality from 31.11% to 87.07% following broflanilide exposure. Docking analyses predicted possible interactions between both proteins and desmethyl-broflanilide (DM-8007), the active metabolite of broflanilide. These results identify *CYP6G8* and *CYP6GX5* as candidate P450 genes associated with broflanilide tolerance under the tested conditions, although their direct biochemical roles remain to be determined.

## Simple Summary

The oriental fruit fly is a serious quarantine pest of fruits and vegetables, and insecticides remain important for its management. Broflanilide is a relatively new insecticide, but little is known about how this pest responds to this compound. We compared gene expression in the midgut and fat body of flies that survived after broflanilide exposure with the gene expression of the control group. Two cytochrome P450 genes showed increased expression. Reducing the expression of either gene increased mortality after broflanilide exposure. Computer simulations also suggested that the proteins encoded by these genes may interact with the active metabolite of broflanilide. These findings identify two candidate genes associated with the response of the oriental fruit fly to broflanilide and provide a basis for further functional studies.

## 1. Introduction

*Bactrocera dorsalis* (Hendel), commonly known as the oriental fruit fly, is a highly invasive and globally important quarantine pest. Its high reproductive capacity, rapid dispersal, and broad host range contribute to its successful spread and establishment in new areas [1]. In China, B. dorsalis was initially restricted to limited areas in the south, but has gradually expanded across most of southern China and more recently spread northward beyond the Yangtze River [2]. The larvae of *B. dorsalis* predominantly damage fruits through internal feeding, subsequently inducing rot or premature drop and reducing their economic value [3]. Consequently, *B. dorsalis* causes extensive damage to various horticultural products and substantial economic losses in China and many other regions worldwide [4,5].

Various technologies have been developed for the management of *B. dorsalis*, while chemical insecticides remain a major component of control systems [6]. However, with their extensive and widespread application, field populations of *B. dorsalis* have developed resistance to a wide spectrum of insecticides, including organophosphates, pyrethroids, and spinosad [6,7]. The rapid development of resistance may reduce the effectiveness of commonly used insecticides and increase the difficulty of managing this pest in production systems. Therefore, insecticides with new modes of action are important for sustainable resistance management. Broflanilide is a novel meta-diamide insecticide classified in IRAC Group 30 and was first registered in China in 2020 [8]. Broflanilide exhibits strong insecticidal activity through contact exposure to treated surfaces [9], and it is metabolized into desmethyl-broflanilide (DM-8007) in insects, which targets the resistant-to-dieldrin (RDL) γ-aminobutyric acid (GABA) receptor and binds to a different site than traditional noncompetitive antagonists [10]. Broflanilide has shown high insecticidal activity against many economically important pests, including Spodoptera frugiperda, Plutella xylostella, and Helicoverpa armigera [11], which can cause substantial crop losses in infested production systems [12,13]. It has also shown toxicity against several tephritid fruit flies, including *B. dorsalis* [14]. To our knowledge, resistance of *B. dorsalis* to broflanilide or other Group 30 insecticides has not yet been reported. These findings indicate that broflanilide may have potential for the future management of *B. dorsalis*, while its molecular responses in this pest remain poorly understood.

Insects can respond to xenobiotic exposure through multiple metabolic and transport processes [15]. Xenobiotic metabolism in insects generally proceeds through three phases. Phase I involves oxidation, reduction, and hydrolysis reactions, and is mainly mediated by cytochrome P450 monooxygenases (P450s) and carboxyl/cholinesterases (CCEs). Phase II involves conjugation reactions mediated by enzymes such as glutathione S-transferases (GSTs) and uridine diphosphate (UDP)-glycosyltransferases (UGTs). In Phase III, metabolites are transported and excreted through ATP-binding cassette (ABC) transporters [16,17,18]. Among these enzyme families, P450s play important roles in xenobiotic metabolism and insecticide resistance [18]. Insecticide exposure can trigger transcriptional induction of P450 genes, as demonstrated by the induced upregulation of *CYP9J45* and *CYP9M10* in *Culex quinquefasciatus*, with similar induction patterns reported in *Musca domestica* and *Drosophila melanogaster* [19,20,21]. In *B. dorsalis*, several P450 genes (e.g., *CYP6D9*, *CYP12C2*, *CYP314A1*) also show altered expression under insecticide stress, such as malathion, abamectin, and beta-cypermethrin [22]. Although the toxicity and sublethal effects of broflanilide have been evaluated in *B. dorsalis* [14], the transcriptional and functional responses of P450 genes to broflanilide in *B. dorsalis* remain largely unknown. Investigating these responses of *B. dorsalis* is therefore essential for understanding its physiological adaptation to broflanilide and for guiding the rational and sustainable application of this insecticide in agricultural systems.

In this study, we performed transcriptomic analyses of the midgut and fat body of the broflanilide-exposed surviving and laboratory control groups. Candidate P450 genes were validated by qRT-PCR, and *CYP6G8* and *CYP6GX5* were further examined using RNAi. Furthermore, molecular docking was employed to predict potential interactions between CYP6G8 and CYP6GX5 and DM-8007, the active metabolite of broflanilide. This study aimed to investigate the transcriptional responses in two representative metabolic tissues (midgut and fat body) of *B. dorsalis* after broflanilide exposure and to identify candidate P450 genes associated with broflanilide tolerance, thereby providing a basis for further understanding the molecular response of *B. dorsalis* to broflanilide and its future rational use in pest management.

## 2. Materials and Methods

### 2.1. Insects and Chemicals

The laboratory population (LAB) of *B. dorsalis* was collected from Guangzhou, Guangdong Province (latitude 23.40° N, longitude 113.22° E) and has been maintained in the laboratory for over 50 generations without exposure to any insecticides. All flies were reared under standardized rearing conditions: 27 ± 0.5 °C, 70 ± 5% relative humidity, and a 16:8 h light: dark photoperiod. Adults were fed a diet consisting of soybean peptone and sucrose at a ratio of 1:3 (*w*/*w*), and water was provided using moistened cotton balls. In this study, a commercial formulation of broflanilide (Cimegra^®^ (BASF Plant Protection (Jiangsu, Nantong), China; country of origin, Singapore), 100 g AI/L, suspension concentrate;) was used as the test insecticide.

### 2.2. Broflanilide Treatment and Selection of Surviving Adults

To obtain broflanilide-exposed surviving adults, the 4-day-old adults of LAB were exposed to broflanilide at its median lethal concentration (LC_50_: 7.559 mg/L, 95% CI: 5.352–12.896 mg/L) using the residual-film method. This LC_50_ value was determined in our previous study [7]. Briefly, five broflanilide concentrations were selected based on preliminary bioassays and prepared by dilution with 0.1% Triton X-100 (Solarbio, Beijing, China). Three replicates of approximately 20 adults were tested at each concentration, mortality was recorded after 24 h, and the LC_50_ and its 95% confidence interval were estimated from the concentration–mortality data using Probit analysis.

Four-day-old adults were selected to minimize age-related physiological variation, and 3–5-day-old adults have been commonly used in previous insecticide bioassays of *B. dorsalis* [23,24]. The residual-film method was used because it is a well-established method for evaluating insecticide susceptibility in adult *B. dorsalis* [23,24], and provides standardized contact exposure.

A residual film was prepared in a 5 L beaker by coating its inner surface with 100 mL of 0.1% Triton X-100 solution containing the LC_50_ concentration. After rotation to ensure uniform coverage, the surface was allowed to air-dry. During exposure, a cotton ball soaked in 10% sucrose solution was placed inside as a food source for adult flies. For the parallel control, adults of the same age were exposed to 0.1% Triton X-100 without broflanilide under the same exposure conditions and for the same 24 h period. After 24 h, surviving adults from the broflanilide-treated group were defined as the broflanilide-exposed surviving group (BFL). Adults from the control group, which were exposed to 0.1% Triton X-100 without broflanilide, were defined as the laboratory control group (LAB). Adults from both groups were collected for RNA-seq and expression analyses.

### 2.3. RNA-Seq

The midgut and fat body were selected as two representative metabolic tissues with complementary roles in xenobiotic processing [25,26]. The midgut and fat body were dissected from adult *B. dorsalis* of both LAB and BFL groups after 24 h of exposure, and these tissues were subsequently used as samples for RNA-seq. Three independent treatment groups were used as three biological replicates for each treatment. Each biological replicate consisted of pooled midguts or fat bodies dissected from 20 adults of mixed-sex individuals. Total RNA was extracted from tissue samples using TRIzol^®^ Reagent (Ambion, Waltham, MA, USA) following standard protocols. RNA quality was assessed using NanoDrop 2000 (Thermo Fisher Scientific, Waltham, MA, USA) for purity (OD260/280: 1.8–2.2) and Agilent 5300 Bioanalyzer (Agilent Technologies, Santa Clara, CA, USA) for integrity (RIN ≥ 6.5) [27]. Poly(A)-enriched mRNA was isolated using oligo magnetic beads (Illumina, San Diego, CA, USA) and fragmented to ~300 base pairs (bp) fragments using fragmentation buffer (Illumina, San Diego, CA, USA). First-strand cDNA was synthesized using SuperScript II Reverse Transcriptase (Invitrogen, Carlsbad, CA, USA) with random hexamer primers, followed by second-strand synthesis. The cDNA fragments were end-repaired, A-tailed, and ligated with Illumina adapters. Library fragments were size-selected (300 bp) using 2% Low Range Ultra Agarose (Biowest, Nuaillé, Pays de la Loire, France) and amplified by PCR (15 cycles) with Phusion DNA Polymerase (New England Biolabs, Ipswich, MA, USA). Final library quantification was performed using Qubit 4.0 Fluorometer (Thermo Fisher Scientific, Waltham, MA, USA), and paired-end sequencing (2 × 150 bp) was conducted on the Illumina NovaSeq 6000 platform (Illumina, San Diego, CA, USA).

### 2.4. Bioinformatic Analyses

Raw sequencing reads were quality-filtered using fastp (v0.20.0) to remove adapter sequences, low-quality bases (Q < 20), and reads with >10% N content [28]. Clean reads were aligned to the reference genome of *B. dorsalis* (GenBank accession: JAGTTC010000000) using HISAT2 (v2.1.0) with default parameters [29]. This reference genome was generated from the same laboratory population used in the present study and therefore closely matched the genetic background of the experimental population. The corresponding genome annotation, containing 20,777 annotated genes, was used for gene quantification [30]. Detailed mapping statistics for all libraries are provided in Supplementary Table S1. Principal component analysis and sample correlation analysis further indicated the consistency and reproducibility among RNA-seq samples (Figure S1). Gene expression levels were quantified using RSEM (RNA-Seq by Expectation-Maximization) (v1.3.1) [31].

Differential expression analysis was performed using DESeq2 (v1.26.0), whereas transcripts per million (TPM) values were used only for expression visualization. *p*-values were adjusted for multiple testing using the Benjamini–Hochberg method, and genes with |log_2_FC| ≥ 1 and a false discovery rate (FDR) < 0.05 were considered differentially expressed [32]. In addition, LFC shrinkage using the apeglm estimator was performed for the selected P450 genes to assess the robustness of their fold-change estimates (Table S2).

For functional annotation, predicted protein sequences were aligned to the NCBI non-redundant protein database using DIAMOND (v0.9.24), and Gene Ontology (GO) terms were assigned accordingly [33]. Kyoto Encyclopedia of Genes and Genomes (KEGG) pathway annotation was performed using KOBAS (v3.0) [34]. Enrichment analysis was performed using Fisher’s exact test with multiple testing correction (Bonferroni, *p* ≤ 0.05).

### 2.5. Quantitative Real-Time PCR (qRT-PCR)

Differentially expressed genes (DEGs) were first screened for genes annotated as cytochrome P450s, from which six P450 genes showing significant differential expression in at least one tissue were selected for qRT-PCR validation. Gene expression levels between broflanilide-exposed surviving and laboratory control groups were compared using the optimal, gene-specific primers described in Table 1. Total RNA samples were reverse transcribed into cDNA using the PrimeScript™ RT reagent Kit with gDNA Eraser (Takara, Kusatsu, Shiga, Japan) to eliminate genomic DNA contamination. The resulting cDNA served as the template for qRT-PCR performed with TB Green^®^ Premix Ex Taq™ II (Takara, Kusatsu, Shiga, Japan). The qRT-PCR reaction was performed in a final volume of 25 μL containing 12.5 μL of TB Green Premix Ex Taq II, 1 μL of cDNA, 1 μL of each primer (10 μM), 0.5 μL of ROX Reference Dye II, and RNase-free water. The cycling program involved an initial step of 95 °C for 30 s, followed by 40 cycles of 95 °C (5 s) and 60 °C (34 s), then 95 °C for 15 s, 60 °C for 1 min, and 95 °C for 15 s. Relative expression levels of target genes were calculated using the 2^−ΔΔCt^ method [35], with 18S rRNA of *B. dorsalis* as the endogenous reference gene for normalization. For candidate gene expression validation, three independent biological replicates were analyzed for each group, and each biological replicate consisted of a pooled sample of 20 midguts or 20 fat bodies of adults.

### 2.6. RNA Interference (RNAi)

The double-stranded RNA (dsRNA) was synthesized using the T7 RiboMAX™ Express RNAi System (Promega Corporation, Madison, WI, USA) according to the manufacturer’s instructions. Briefly, target gene fragments of approximately 400 bp were amplified from cDNA of *B. dorsalis* using gene-specific primers (Table 1). PCR products were gel-purified using the AxyPrep DNA Gel Extraction Kit (Axygen Biosciences, Union City, CA, USA), then cloned into pGEM^®^-T vectors (Promega, Madison, WI, USA) for sequencing verification. T7 promoter sequences (5′-TAATACGACTCACTATAGG-3′) were incorporated via PCR using verified plasmid clones. The amplified products were purified through ethanol precipitation and served as templates for in vitro transcription to generate dsRNA. The synthesized dsRNA was treated with RNase and DNase, precipitated with isopropanol, washed with 70% ethanol, resuspended in nuclease-free water, and stored at −20 °C.

For RNAi, dsRNA targeting two upregulated genes (*CYP6G8* and *CYP6GX5*) and dsGFP were separately complexed with Star Polycation (SPC) nanocarrier at a 1:1 mass ratio (*w*/*w*) and incubated on ice for 15 min to allow nanoparticle formation [36]. Then the mixture was applied uniformly to the solid medium feed [7]. The stability of the dsRNA–SPC complex on the solid medium during the 24 h feeding period was not directly evaluated in the present study. Four-day-old LAB adults were fed either target-gene or *GFP* dsRNA complexes. The amount of dsRNA supplied through the feeding medium corresponded to approximately 1 μg per fly. At 24 h post-feeding, whole insects were collected for the detection of the efficiency of RNAi-mediated gene knockdown. For *CYP6G8*, 10 independent biological replicates were initially prepared for each treatment, and 6 independent biological replicates were initially prepared for each treatment in the *CYP6GX5* knockdown assay. Only RNA samples with A260/A280 = 1.8–2.2 and RNA concentration ≥ 200 ng/μL were included in subsequent qRT-PCR analysis, and the number of final samples is provided in the figure legend. For *CYP6G8*, one dsGFP replicate and three dsCYP6G8 replicates were excluded based on these criteria. For *CYP6GX5*, no dsGFP replicate and two dsCYP6GX5 replicates were excluded.

To evaluate the effect of gene silencing on broflanilide susceptibility, adults were exposed to broflanilide at the LC_50_ concentration using the residual-film method after 24 h of dsRNA feeding. For *CYP6G8*, each treatment was replicated five times, whereas three replicates were established for each treatment in the *CYP6GX5* experiment, and each replicate contained about 20 adult flies. In parallel, dsGFP-treated and target-dsRNA-treated adults were exposed to 0.1% Triton X-100 as corresponding control treatments without broflanilide. One replicate in the dsGFP group of the *CYP6G8* bioassay was excluded because excessive moisture from the cotton ball caused abnormally high mortality unrelated to the insecticide treatment. After a 24-h exposure period, mortality was assessed. Adults who showed no movement within 30 s of gentle brush probing were classified as dead. Bioassays were considered valid when mortality in the control group was below 20%.

### 2.7. Protein Structure Prediction and Molecular Docking Analysis

The 3D protein structures of CYP6G8 and CYP6GX5 were predicted using the platform AlphaFold3 [37], and their stereochemical qualities were assessed using a Ramachandran plot analysis on the PROCHECK server [38]. The molecular structure of desmethyl-broflanilide (compound CID: 87288706) was obtained from the PubChem database [39], and the potential binding pockets within the predicted structures of CYP6G8 and CYP6GX5 were identified using the DoGSite3 tool integrated within the ProteinsPlus web server [40]. Docking was performed using AutoDock Vina (v1.2.5) [41], with the grid box specifically centered on the predicted binding pocket. The docking pose with the lowest binding energy was selected, and protein-ligand interactions such as hydrogen bonds, halogen bonds, and hydrophobic contacts were analyzed using PyMOL (v1.8) (Schrödinger, LLC, New York, NY, USA) and Protein-Ligand Interaction Profiler (PLIP 2025) [42].

### 2.8. Graphical Presentation and Statistical Analysis

All graphs in this study were generated using OriginPro 2022 software (OriginLab Corporation, Northampton, MA, USA). The efficiency of RNAi and mortality data were analyzed with IBM SPSS Statistics 26 (IBM Corp., Armonk, NY, USA). For comparisons of gene expression and other quantitative data, differences between two groups were evaluated using independent samples *t*-tests. Welch’s *t*-test was used when variances were unequal. All data are presented as the mean ± standard error of the mean (SEM). Statistical significance was set at * *p* < 0.05, ** *p* < 0.01, and *** *p* < 0.001. The silencing efficiency of RNA interference (RNAi) was calculated using Equation (1).

Silencing efficiency (%) = (1 − Relative expression of RNAi group/Relative expression of control group) × 100%(1)

## 3. Results

### 3.1. BFL and LAB Show Transcriptomic Differences in the Midgut and Fat Body

**Figure 1:**
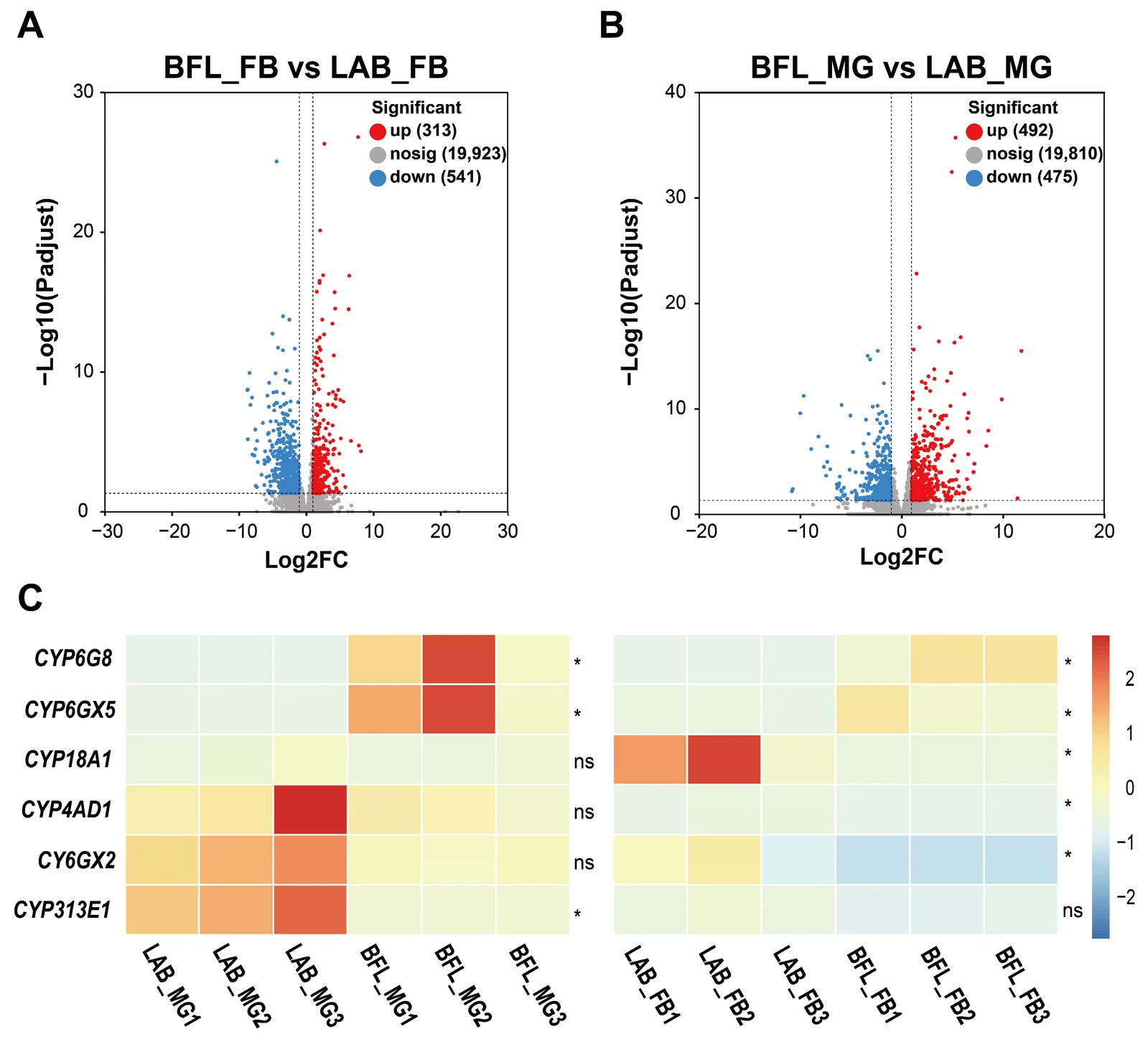
Comparative transcriptomic analysis of midgut and fat body in broflanilide-exposed surviving and laboratory control groups of *Bactrocera dorsalis*. Volcano plots of differentially expressed genes (DEGs) from (**A**) the fat body and (**B**) the midgut. The *x*-axis represents the log_2_ fold change (FC) (broflanilide-exposed surviving/laboratory control), and the y-axis shows the −log_10_ of the adjusted *p*-value. Red and blue dots denote significantly upregulated (log_2_FC ≥ 1, adjusted *p*-value < 0.05) and downregulated (log_2_FC ≤ −1, adjusted *p*-value < 0.05) genes, respectively, while gray dots represent non-significant genes. Dashed lines indicate the thresholds for statistical significance and fold change. (**C**) Heatmap of the expression patterns of candidate P450 genes in broflanilide-exposed surviving (BFL) and laboratory control (LAB) groups of *Bactrocera dorsalis*. Each row represents a gene, and each column represents a biological replicate. Expression levels are shown as Z-scores of normalized counts, with red indicating high expression and blue indicating low expression relative to the mean across all samples. * indicates a statistically significant difference; ns indicates no significant difference.

Comparative transcriptomic analysis between BFL and LAB identified 313 upregulated and 541 downregulated genes in fat bodies (Figure 1A), with an additional 492 upregulated and 475 downregulated genes in midgut (Figure 1B). Among the P450 genes selected for further validation, *CYP6G8* and *CYP6GX5* showed significant upregulation in both midgut and fat body tissues of BFL relative to LAB, while *CYP18A1*, *CYP4AD1* and *CYP6GX2* were specifically downregulated in fat body and *CYP313E1* exhibited midgut-specific downregulation (Figure 1C).

**Figure 2:**
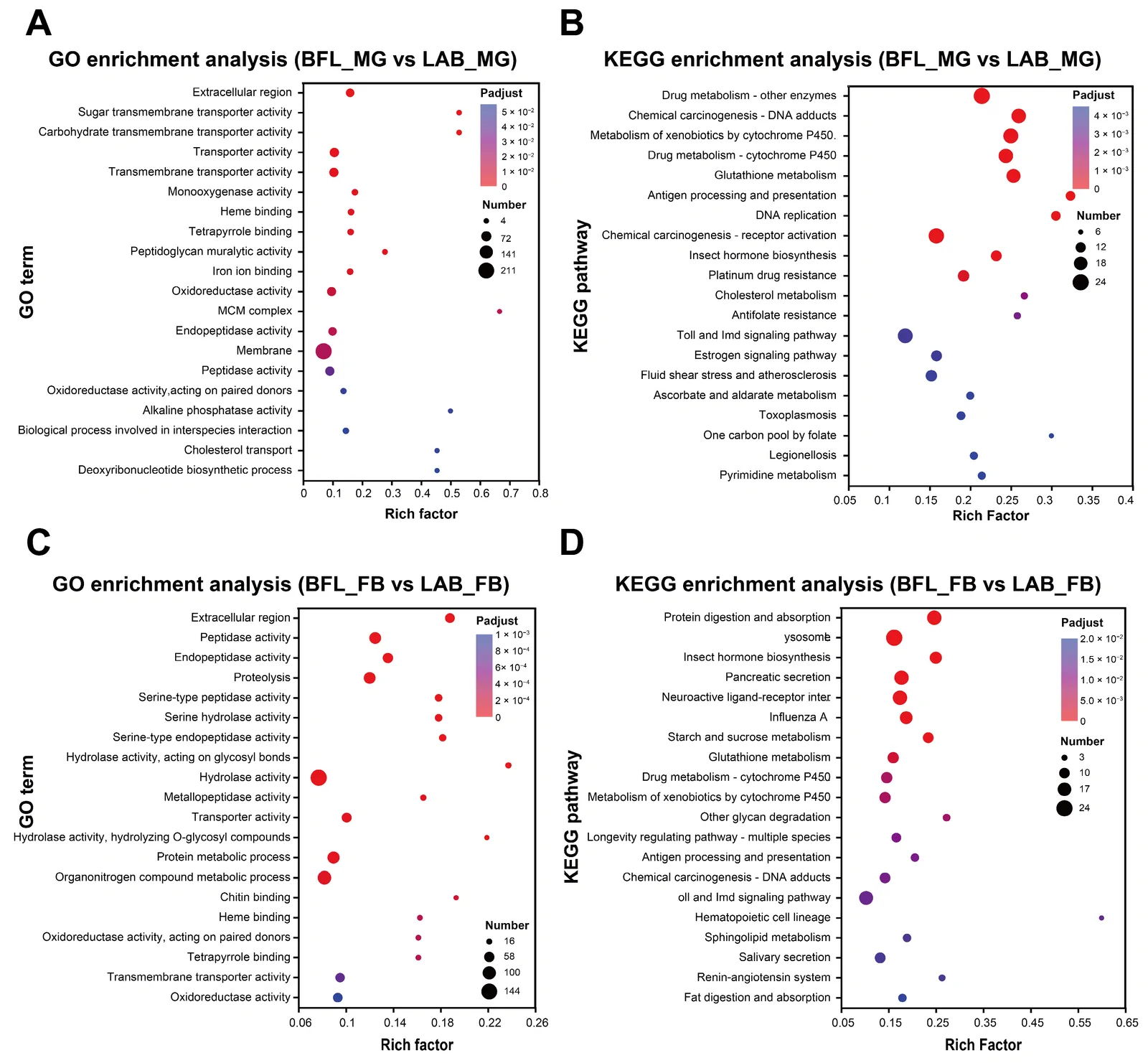
Functional enrichment of differentially expressed genes (DEGs) from midgut and fat body tissues in *B. dorsalis*. (**A**,**C**) Gene Ontology (GO) terms significantly enriched in DEGs from (**A**) midgut and (**C**) fat body. (**B**,**D**) Kyoto Encyclopedia of Genes and Genomes (KEGG) pathways significantly enriched in DEGs from (**B**) midgut and (**D**) fat body. The *x*-axis represents the rich factor, bubble size represents the number of DEGs assigned to each term, and bubble color represents the adjusted *p*-value.

In the midgut, GO enrichment analysis specifically highlighted enriched terms for extracellular region, transporter activity, monooxygenase activity, heme binding, and membrane (Figure 2A). KEGG pathway analysis revealed a highly significant enrichment in “Metabolism of xenobiotics by cytochrome P450”, “Drug metabolism—cytochrome P450”, and “Glutathione metabolism” (Figure 2B). In the fat body, GO terms were primarily enriched in extracellular processes, proteolytic activities, and metabolic functions (Figure 2C). KEGG analysis showed significant enrichment of pathways including “Insect hormone biosynthesis” and “Protein digestion and absorption”, “Glutathione metabolism”, and “Drug metabolism—cytochrome P450” (Figure 2D).

### 3.2. CYP6G8 and CYP6GX5 Show Distinct Tissue-Specific Upregulation Patterns

**Figure 3:**
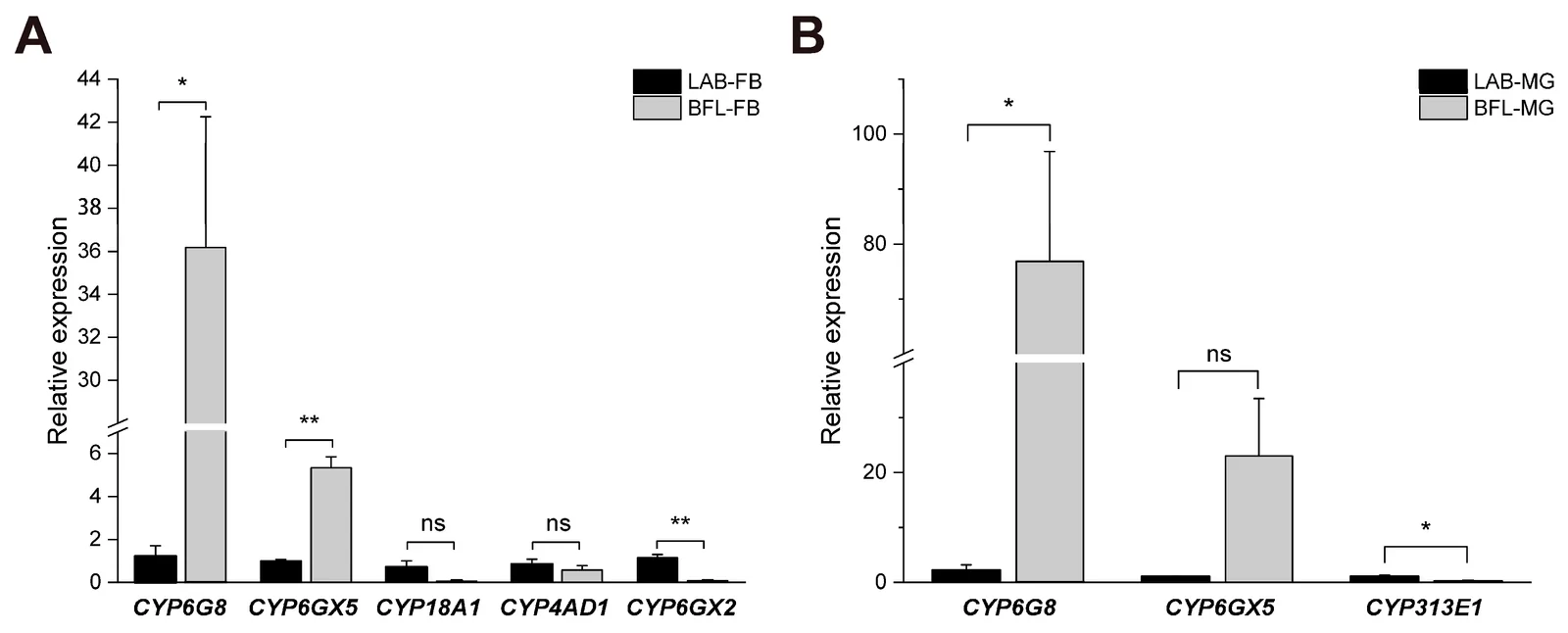
Tissue-specific expression of candidate P450 genes following broflanilide exposure in *B. dorsalis*. Relative expression levels of candidate genes were validated by quantitative real-time PCR (qRT-PCR) in (**A**) fat body and (**B**) midgut of broflanilide-exposed surviving (BFL) and laboratory control (LAB) groups. Data are presented as mean ± SEM (*n* = 3). Statistical comparisons between BFL and LAB groups for each gene and tissue were performed using independent samples t-tests with Welch’s correction applied when variances were unequal. ** *p* < 0.01, * *p* < 0.05, ns, not significant.

The qRT-PCR results generally showed expression trends consistent with the RNA-seq analysis. Among the upregulated genes, *CYP6G8* showed significant upregulation in both midgut (*t* = −3.728, df = 3.016, *p* = 0.033) and fat body (*t* = −5.729, df = 2.026, *p* = 0.028) of BFL. In contrast, *CYP6GX5* showed significant upregulation exclusively in the fat body of BFL (*t* = −8.325, df = 4, *p* = 0.00114), whereas an upward trend was observed in the midgut, but the difference was not statistically significant. Downregulated genes displayed pronounced tissue-specific expression patterns. In the fat body of BFL, *CYP6GX2* (*t* = 6.607, df = 4, *p* = 0.003) showed significant downregulation. In the midgut of BFL, *CYP313E1* (*t* = 3.523, df = 4, *p* = 0.024) was significantly downregulated. Additionally, *CYP18A1* and *CYP4AD1* also showed decreasing trends in the fat body, although the differences were not statistically significant (Figure 3).

### 3.3. Silencing of CYP6G8 and CYP6GX5 Increases Susceptibility to Broflanilide

**Figure 4:**
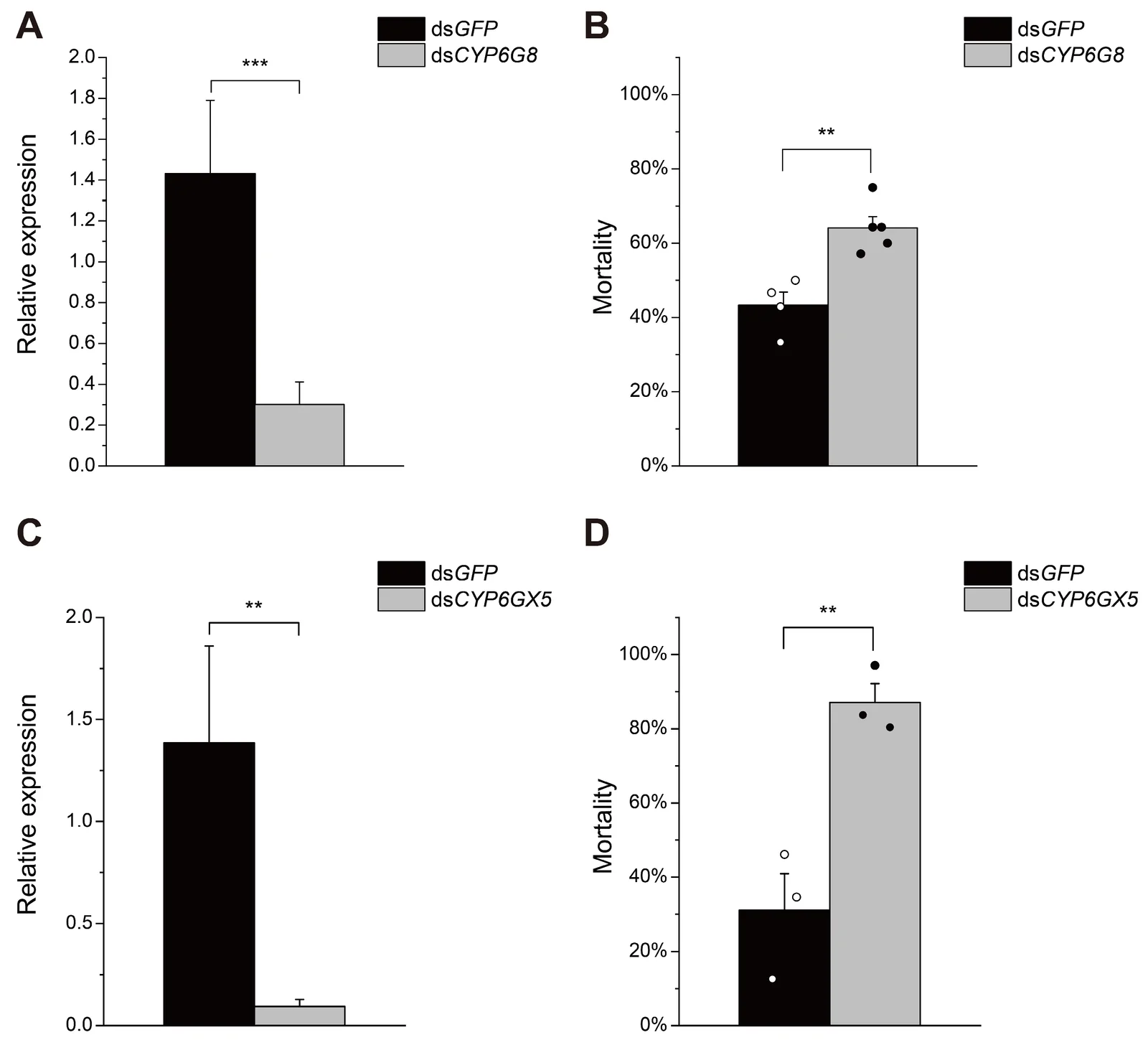
Functional validation of candidate P450 genes through RNA interference. (**A**) Knockdown efficiency of *CYP6G8* (dsGFP, *n* = 9; dsCYP6G8, *n* = 7) and (**B**) mortality after broflanilide exposure following *CYP6G8* silencing (dsGFP, *n* = 4; dsCYP6G8, *n* = 5). (**C**) Knockdown efficiency of *CYP6GX5* (dsGFP, *n* = 6; dsCYP6GX5, *n* = 4) and (**D**) mortality after broflanilide exposure following *CYP6GX5* silencing (dsGFP, *n* = 3; dsCYP6GX5, *n* = 3). Data are presented as mean ± SEM, and dots represent individual biological replicates. Statistical significance was determined by independent-samples *t*-tests, with Welch’s correction applied when variances were unequal, comparing each dsRNA group to the dsGFP control. *** *p* < 0.001, ** *p* < 0.01.

Silencing efficiencies for *CYP6G8* (78.9%) (*t* = 2.998, df = 9.444, *p* = 0.014) and *CYP6GX5* (93.2%) (*t* = 2.715, df = 5.053, *p* = 0.042) were confirmed by qRT-PCR at 24 h post-treatment, relative to the dsGFP control. The dsRNA treatment significantly increased mortality after broflanilide exposure compared to the parallel controls. Specifically, the mortality in the dsCYP6G8 group reached 64.14% ± 3.03%, significantly exceeding that of the dsGFP control (43.21% ± 3.60%) (*t* = −4.478, df = 7, *p* = 0.003). *CYP6GX5* silencing elevated mortality to 87.07% ± 5.09%, a pronounced increase from the 31.11% ± 9.83% observed in the control group (*t* = −5.056, df = 3, *p* = 0.015). Both comparisons were statistically significant (Figure 4). No mortality was observed in either dsGFP-treated or target-dsRNA-treated adults when exposed to the 0.1% Triton X-100 control treatment without broflanilide. Thus, no detectable mortality caused by gene silencing alone was observed under these experimental conditions.

### 3.4. Molecular Docking Predicts Potential Interactions of CYP6G8 and CYP6GX5 with DM-8007

**Figure 5:**
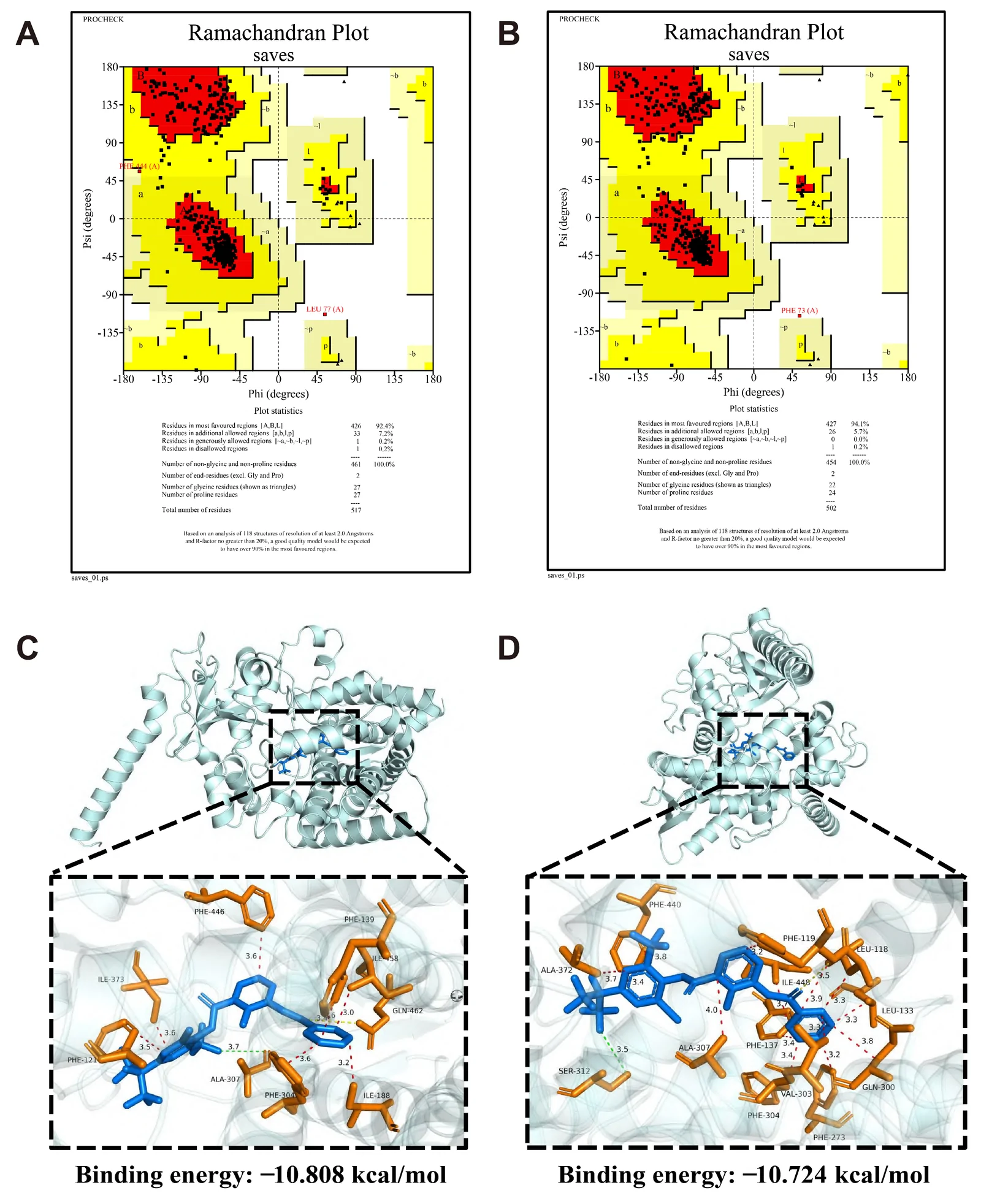
Predicted interactions of CYP6G8 and CYP6GX5 with desmethyl-broflanilide based on molecular docking. (**A**) Ramachandran plot of the CYP6G8 3D structure; (**B**) Ramachandran plot of the CYP6GX5 3D structure; (**C**) Binding mode of desmethyl-broflanilide within the pocket of CYP6G8. (**D**) Binding mode of desmethyl-broflanilide within the pocket of CYP6GX5. The ligand is shown in blue, interacting amino acid residues are shown in orange, and protein structures are shown in light blue. Yellow, red, and green dashed lines represent hydrogen bonds, hydrophobic interactions, and halogen bonds, respectively.

Ramachandran plot analysis indicated acceptable stereochemical quality of the predicted protein models, with 92.4% (CYP6G8) and 94.1% (CYP6GX5) of residues located in favoured regions (Figure 5A,B). Docking simulations indicated that desmethyl-broflanilide (DM-8007) could be accommodated within the predicted binding pockets of both CYP6G8 and CYP6GX5. The predicted binding energies were −10.808 kcal/mol for CYP6G8 and −10.724 kcal/mol for CYP6GX5.

In the predicted CYP6G8–DM-8007 binding pose, a hydrogen bond with GLN-462 and a halogen bond with ALA-307 were identified. Additionally, several hydrophobic contacts including PHE-121, PHE-139, PHE-188, PHE-304, ILE-373, PHE-446, and PHE-458 were also predicted around DM-8007 within the binding pocket (Figure 5C). For CYP6GX5, the predicted binding pose included a hydrogen bond with PHE-119 and halogen bonds involving SER-312, together with additional contacts involving LEU, PHE, and GLN residues (Figure 5D).

## 4. Discussion

The rapid spread of *B. dorsalis* has caused considerable economic losses in fruit and vegetable production worldwide, and chemical insecticides remain important for its management. Broflanilide has shown insecticidal activity against *B. dorsalis*, but the molecular responses of this pest to broflanilide remain poorly understood. In the present study, comparative transcriptomic analysis of BFL and LAB revealed distinct expression patterns in the midgut and fat body, with several P450-associated pathways enriched in both tissues. Further qRT-PCR and RNAi analyses identified *CYP6G8* and *CYP6GX5* as candidate genes associated with broflanilide tolerance under the tested conditions. Molecular docking additionally predicted possible interactions between the two CYP proteins and DM-8007. Together, these results provide evidence for the involvement of *CYP6G8* and *CYP6GX5* in the response of *B. dorsalis* to broflanilide, although their direct biochemical functions remain to be determined.

The midgut and fat body are important metabolic tissues in insects and showed different transcriptomic patterns between BFL and LAB in the present study. In the midgut, the significant enrichment of GO terms such as “heme binding” and “monooxygenase activity”, alongside KEGG pathways like “Metabolism of xenobiotics by cytochrome P450” and “Drug metabolism- cytochrome P450”, suggests that P450-associated processes were involved in the observed transcriptional differences [43]. In the fat body, beyond detoxification pathways, enriched pathways were also associated with insect hormone biosynthesis, protein digestion and absorption, and nutrient metabolism, indicating a broader physiological response. Similar changes in metabolic processes have been reported in *Bombyx mori* following insecticide exposure [44]. Although the two tissues showed distinct transcriptomic patterns, P450-associated pathways were consistently enriched in both tissues. These results supported our focus on candidate P450 genes for subsequent expression and functional analyses.

Studies on P450 responses to broflanilide and related diamide insecticides remain limited in Tephritidae and Diptera. Previous studies of broflanilide in tephritid flies, including *B. dorsalis*, have mainly focused on toxicity and sublethal effects [14]. However, P450 involvement in responses or resistance to diamide insecticides has been reported in other insects, including *Spodoptera frugiperda*, *Spodoptera exigua*, and *Tuta absoluta* [45,46,47]. These studies suggest that P450 genes can be involved in responses or resistance to different diamide insecticides, although the roles of individual P450s may vary among insect species and insecticides.

In the present study, six P450 genes showing significant differential expression in at least one tissue were selected for qRT-PCR validation. Several P450 genes, such as *CYP6GX2* and *CYP313E1,* exhibited tissue-specific downregulation trends in either the midgut or fat body, which is a common feature of the insect toxicological response. In addition, *CYP18A1* and *CYP4AD1* were significantly downregulated in the fat body according to RNA-seq, whereas qRT-PCR showed similar decreasing trends without statistical significance. These differences may result from differences in quantification methods, biological variation among replicates, and differences in detection sensitivity and statistical power between RNA-seq and qRT-PCR. In contrast to these downregulated genes, RNA-seq showed that both *CYP6G8* and *CYP6GX5* were significantly upregulated in the midgut and fat body. qRT-PCR further confirmed significant upregulation of *CYP6G8* in both tissues and of *CYP6GX5* in the fat body, and *CYP6GX5* showed a nonsignificant upward trend in the midgut, which suggested that these two genes might be involved in the response of *B. dorsalis* to broflanilide exposure. Therefore, they were selected as the primary candidates for further functional validation. RNAi-mediated silencing of either *CYP6G8* or *CYP6GX5* increased mortality following broflanilide exposure, supporting their contribution to broflanilide tolerance under the tested conditions.

Previous studies have also reported different expression patterns of *CYP6G8* and *CYP6GX5* under other insecticide conditions. *CYP6G8* was significantly downregulated in the malathion-resistant population of *B. dorsalis*, which contrasts with its increased expression in BFL in the present study [48]. This differential expression suggests that the regulation of *CYP6G8* may depend on the insecticide and physiological context. Its increased expression in BFL may therefore reflect not only a detoxification-related response but also a broader stress or adaptive response associated with the treatment. In contrast, *CYP6GX5* was previously reported to be upregulated in the lambda-cyhalothrin-resistant population of *B. dorsalis* [7], consistent with its increased expression in BFL in the present study, suggesting that *CYP6GX5* may be involved in responses to more than one insecticide. Overall, the expression and RNAi results support the involvement of *CYP6G8* and *CYP6GX5* in broflanilide tolerance, although they do not demonstrate direct metabolism of broflanilide by either CYP protein.

Broflanilide is considered a pro-insecticide, as its insecticidal activity mainly depends on conversion to the active metabolite DM-8007, which targets the insect GABA receptor [10]. The increased mortality observed after silencing *CYP6G8* or *CYP6GX5* is important for interpreting their possible roles in this process. If these P450s mainly converted broflanilide into DM-8007, their silencing would be expected to reduce the formation of the active metabolite and therefore decrease toxicity. This is inconsistent with the increased mortality observed after RNAi treatment. Therefore, one possible explanation is that these P450s may participate in the further metabolism or detoxification of DM-8007 or related compounds. However, this remains a hypothesis.

Therefore, molecular docking was specifically performed with DM-8007 rather than broflanilide to explore its potential structural interactions with CYP6G8 and CYP6GX5. The results predicted possible interactions of DM-8007 with both CYP proteins. However, molecular docking only provides predicted binding poses and energies and cannot demonstrate physical binding or catalytic metabolism. Moreover, the predicted binding energies of CYP6G8 and CYP6GX5 were very similar, and no other P450 proteins or negative controls were included for comparison. Therefore, the specificity of these predicted interactions cannot be determined from the present analysis. Moreover, the AlphaFold3 structures used for docking were also computationally predicted and have not been experimentally validated. Further in vitro metabolic assays using recombinant CYP proteins and both broflanilide and DM-8007 as substrates are required to determine the actual biochemical roles of CYP6G8 and CYP6GX5.

Although the present results support the involvement of *CYP6G8* and *CYP6GX5* in the response of *B. dorsalis* to broflanilide exposure, several limitations should be acknowledged. Because the BFL group consisted of adults surviving 24 h of broflanilide exposure, the observed expression differences cannot completely distinguish exposure-related changes from pre-existing differences among surviving individuals. In addition, only one concentration (LC_50_) and one sampling time point (24 h) were examined, limiting the assessment of temporal and concentration-dependent responses. The use of the commercial formulation Cimegra^®^ without a formulation-matched control also means that possible effects of formulation additives cannot be completely excluded. These additives may affect cuticular penetration, the amount of active ingredient entering the insect, and physiological or P450-related responses [49,50]. Therefore, the observed transcriptional differences should be interpreted as being associated with the treatment and survival rather than attributed exclusively to broflanilide itself. Future studies using multiple exposure conditions and analytical-grade broflanilide or an appropriate formulation control would help distinguish these effects more clearly.

In addition, the RNAi assays were designed as an initial functional test at the LC_50_ concentration. Therefore, they show altered mortality under this condition but cannot show changes in susceptibility across concentrations. Tissue-specific knockdown efficiency also remains to be determined. The dsGFP treatment served as the negative control under the same dsRNA–SPC delivery and feeding conditions, and no mortality was observed in the absence of broflanilide. However, a completely untreated control was not included, limiting the evaluation of possible background effects of the delivery system. In addition, the relatively small number of replicates in the *CYP6GX5* mortality assay (*n* = 3) also limits the statistical robustness of this result, and additional biological replicates will be included in further validation.

This study identified *CYP6G8* and *CYP6GX5* as candidate P450 genes associated with the response of *B. dorsalis* to broflanilide exposure. Transcriptomic analyses of the midgut and fat body indicated distinct tissue-specific expression patterns between BFL and LAB, with P450-associated pathways enriched in both tissues. RNAi assays further supported that these genes contribute to broflanilide tolerance under the conditions tested, and molecular docking predicted possible interactions between DM-8007 and two P450 proteins. From an applied perspective, *CYP6G8* and *CYP6GX5* may serve as candidate genes for further population-level investigations of broflanilide response. Future studies should determine whether the expression of these genes is consistently associated with susceptibility to broflanilide across geographical populations and further clarify their biochemical functions through direct metabolic assays. Overall, these findings provide a molecular basis for further understanding broflanilide response in *B. dorsalis* and may contribute to resistance risk assessment and rational use of insecticides.

## Figures and Tables

**Table 1:** Primer sequences used for qRT-PCR and dsRNA synthesis of candidate P450 genes.

Application	Primer Name	Primer Sequence (5′-3′)	Expected Amplicon Size (bp)	Amplification Efficiency	R^2^
qRT-PCR	*CYP6G8*-F	CTAGGCGTACATCGTGACCC	114	96.62%	0.9844
	*CYP6G8*-R	GCCGAAAGGAAAGTAGGCCA			
	*CYP6GX5*-F	ACCCTCAGGCTTTTCTCGGT	194	94.88%	0.9898
	*CYP6GX5*-R	CAACCACACTTTGCTACTTGGC			
	*CYP4AD1*-F	GTACGAACTGAAGTCGCTGC	200	96.63%	0.9915
	*CYP4AD1*-R	CGTTCGTTCGTGTTTTCAGTCT			
	*CYP18A1*-F	TTGCTAACGCGACAATGCAC	185	105.36%	0.9981
	*CYP18A1*-R	TGCTTCTCGTTGCCCATGAA			
	*CYP6GX2*-F	TGCTGGTCAGCTATCTACGC	199	91.48%	0.9901
	*CYP6GX2*-R	ACGGCTATCGGTTTGGTGAA			
	*CYP313E1*-F	ATTACGGCACGCACGAAAAC	153	97.83%	0.9891
	*CYP313E1*-R	TGTCAACCATACCCCGAAAAC			
	*18S*-F	GCGAGAGGTGAAATTCTTGG	191	102.29%	0.9966
	*18S*-R	CGGGTAAGCGACTGAGAGAG			
RNAi	dsCYP6G8-F	TACTCAACAAGCCAGCGTT	389	-	-
	dsCYP6G8-R	GCCGTTTGGATTACTAAGACT			
	dsCYP6G8-T7F	TAATACGACTCACTATAGGTACTCAACAAGCCAGCGTT	427	-	-
	dsCYP6G8-T7R	TAATACGACTCACTATAGGGCCGTTTGGATTACTAAGACT			
	dsCYP6GX5-F	CAACAAATGGACGAAGCTCT	338	-	-
	dsCYP6GX5-R	TGTTCTTCGCGTGTATGCAA			
	dsCYP6GX5-T7F	TAATACGACTCACTATAGGCAACAAATGGACGAAGCTCT	376	-	-
	dsCYP6GX5-T7R	TAATACGACTCACTATAGGTGTTCTTCGCGTGTATGCAA			
	dsGFP-F	CACAAGTTCAGCGTGTCCG	420	-	-
	dsGFP-R	GTTCACCTTGATGCCGTTC			
	dsGFP-T7F	TAATACGACTCACTATAGGCACAAGTTCAGCGTGTCCG	458	-	-
	dsGFP-T7R	TAATACGACTCACTATAGGGTTCACCTTGATGCCGTTC			

## Data Availability

The RNA-Seq raw data underlying this study have been uploaded to the Sequence Read Archive (SRA) under NCBI BioProject PRJNA1395032, with accession numbers SRR36634145-SRR36634156, and will be publicly released upon publication.

## Supplementary Materials

The following supporting information can be downloaded at: https://www.mdpi.com/article/10.3390/insects17090950/s1, Figure S1: Principal component analysis and sample correlation analysis of RNA-seq samples. (A) PCA of RNA-seq samples based on gene expression profiles. (B) Heatmap showing correlations among RNA-seq samples.; Table S1: Summary of sequencing and mapping statistics for all RNA-seq libraries; Table S2: Comparison of original and apeglm-shrunken log2 fold-change estimates for six selected P450 genes in the midgut and fat body of *Bactrocera dorsalis*.
