# Supplementary material for: The Upregulation of Two CYP6 Genes Is Associated with the Response to Broflanilide in Bactrocera dorsalis (Hendel, 1912) (Diptera: Tephritidae)
Source: Insects. 2026 Sep 11;17(9):950. doi: 10.3390/insects17090950 (PMC13607031; doi:10.3390/insects17090950)
Supplement: Supplementary file 1 [file insects-17-00950-s001.zip › insects-4497439-supplementary.pdf]

Table S1 Summary of sequencing and mapping statistics for all RNA-seq libraries

Sample	Total reads	Total mapped reads	Mapping rate	Multiple mapped reads	Multiple mapping rate (%)	Uniquely mapped reads	Unique mapped rate (%)
BFL_FB1	*97154640*	42842698	85.83%	2638859	5.29%	40203839	80.54%
BFL_FB2	*51089122*	38234442	84.42%	2662655	5.88%	35571787	78.54%
BFL_FB3	*51522982*	36599322	69.78%	2197422	4.19%	34401900	65.59%
BFL_MG1	*40172286*	33685845	83.85%	1939393	4.83%	31746452	79.03%
BFL_MG2	*39052590*	27915649	71.48%	1570794	4.02%	26344855	67.46%
BFL_MG3	*55926196*	39272081	70.22%	2064418	3.69%	37207663	66.53%
LAB_FB1	*47930496*	41964570	43.19%	2043527	2.1%	39921043	41.09%
LAB_FB2	*51883252*	9328795	18.26%	474943	0.93%	8853852	17.33%
LAB_FB3	*51119052*	17747827	34.45%	1010172	1.96%	16737655	32.49%
LAB_MG1	*47930496*	27863690	58.13%	1412158	2.95%	26451532	55.19%
LAB_MG2	*51883252*	20867115	40.22%	899915	1.73%	19967200	38.48%
LAB_MG3	*51119052*	11789126	23.06%	502757	0.98%	11286369	22.08%

Table S2 Comparison of original and apeglm-shrunken log2 fold-change estimates for six selected P450 genes in the midgut and fat body of *Bactrocera dorsalis*

Gene	Tissue	Original log2FC	Shrunken log2FC
CYP6G8	Fatbody	6.3175	6.1529
	Mid gut	11.8402	13.1054
CYP6GX5	Fatbody	7.8274	7.0556
	Mid gut	6.4757	5.6920
CYP4AD1	Fatbody	-2.8652	-2.5113
	Mid gut	–0.7925	–0.3754
CYP18A1	Fatbody	-3.8880	-3.5874
	Mid gut	0.1501	0.0227
CYP6GX2	Fatbody	-2.2745	-2.1135
	Mid gut	–0.6892	–0.6232
CYP313E1	Fatbody	-3.7157	-0.1967
	Mid gut	–2.1275	–1.7672

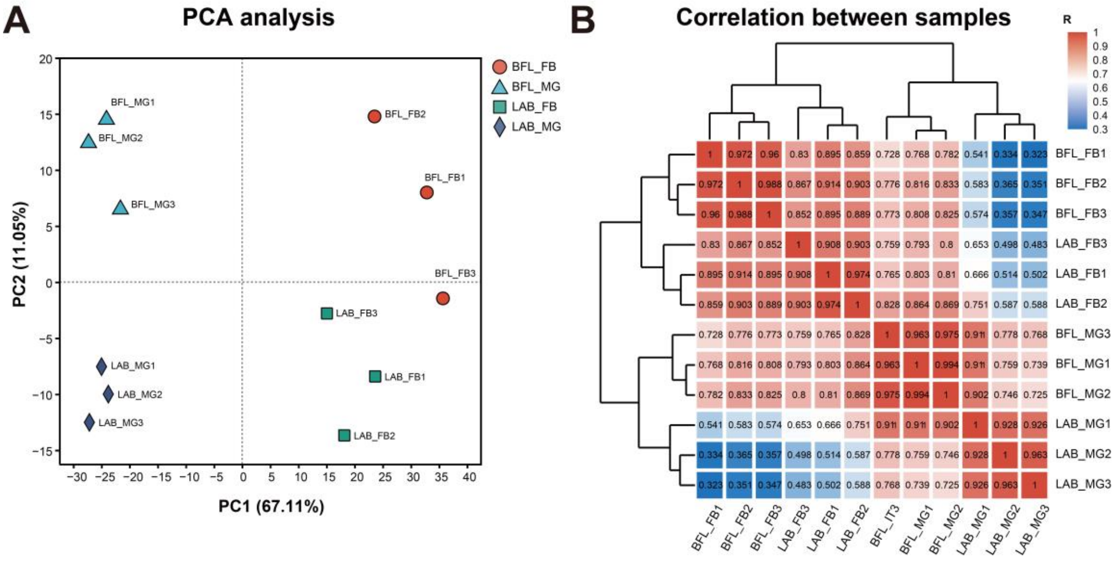

Figure S1. Principal component analysis and sample correlation analysis of RNA-seq samples. (A) PCA of RNA-seq samples based on gene expression profiles. (B) Heatmap showing correlations among RNA-seq samples.
